# Pit Latrine Emptying Behavior and Demand for Sanitation Services in Dar Es Salaam, Tanzania

**DOI:** 10.3390/ijerph120302588

**Published:** 2015-02-27

**Authors:** Marion W. Jenkins, Oliver Cumming, Sandy Cairncross

**Affiliations:** 1 Department of Civil and Environmental Engineering, University of California Davis, One Shields Ave., Davis, CA 95616, USA; 2 Department of Disease Control, Faculty of Infectious and Tropical Diseases, London School of Hygiene and Tropical Medicine, Keppel Street, London WC1E 7HT, UK; E-Mails: oliver.cumming@lshtm.ac.uk (O.C.); sandy.cairncross@lshtm.ac.uk (S.C.)

**Keywords:** urban sanitation, fecal sludge management, willingness to pay for emptying services, Gulper manual de-sludging pump, latrine replacement, emptying services, emptying frequency, empting costs, pit additives, affordability

## Abstract

Pit latrines are the main form of sanitation in unplanned areas in many rapidly growing developing cities. Understanding demand for pit latrine fecal sludge management (FSM) services in these communities is important for designing demand-responsive sanitation services and policies to improve public health. We examine latrine emptying knowledge, attitudes, behavior, trends and rates of safe/unsafe emptying, and measure demand for a new hygienic latrine emptying service in unplanned communities in Dar Es Salaam (Dar), Tanzania, using data from a cross-sectional survey at 662 residential properties in 35 unplanned sub-wards across Dar, where 97% had pit latrines. A picture emerges of expensive and poor FSM service options for latrine owners, resulting in widespread fecal sludge exposure that is likely to increase unless addressed. Households delay emptying as long as possible, use full pits beyond what is safe, face high costs even for unhygienic emptying, and resort to unsafe practices like ‘flooding out’. We measured strong interest in and willingness to pay (WTP) for the new pit emptying service at 96% of residences; 57% were WTP ≥U.S. $17 to remove ≥200 L of sludge. Emerging policy recommendations for safe FSM in unplanned urban communities in Dar and elsewhere are discussed.

## 1. Introduction

Pit latrines persist as the main form of sanitation among the urban poor in Africa and many other developing regions, especially where unplanned or informal settlements dominate the urban landscape [1,2,3,4,5]. When pit latrines fill, the options are limited to latrine replacement or pit-emptying [6,7]; where space is limited emptying may be the only option. Thus in high density areas, safe management of full pits is critical to maintaining access to safe sanitation and a necessary measure for improving public health.

Despite an obvious need for safe fecal sludge emptying, treatment, and disposal in such settings, there has been little systematic investigation of the availability and access to safe fecal sludge management (FSM) services for pit latrine owners in developing country cities. Similarly, there has been limited systematic study of households’ emptying and replacement behaviors to understand the factors associated with unsafe emptying and the challenges and constraints of poor urban residents in managing full latrines. The extent to which demand for, and supply of, pit emptying services exists and involves unsafe methods is critical to assess sanitation public health risks and encourage safe FSM practices. This kind of information is also needed for the design of demand-responsive and sustainable city-wide sanitation development plans and investment decisions. Such information and understanding is likely to become increasingly important in light of the latest global recommendations for post-2015 development goals and targets for sanitation to include progressive achievement of safe fecal sludge management in urban areas [8]. 

In Dar Es Salaam (Dar), Tanzania’s largest city, a systematic assessment of residential sanitation facilities in unplanned settlements found that nearly all households (97%) used pit latrines but access to hygienically safe pit emptying services was low [9]. Building on that earlier assessment with data collected in the same survey, we examine local perceptions of sanitation conditions and household FSM behaviors and expenditures in the context of service availability, affordability, and attitudes towards hygienic emptying services. We use this information to estimate the size of the current market in pit emptying services and rates of unsafe emptying. Finally, we assess household demand in Dar’s unplanned areas for a new hygienic emptying service based on the Gulper, a manually operated sludge extraction pump designed for pit latrines in dense areas [7,10]. The aim of the study is to provide behavioral understanding of demand for improved sanitation services in unplanned urban areas to support development of safe FSM services where such services are currently inadequate or lacking.

## 2. Methods and Materials 

Data come from a cross-sectional survey in June 2008 at 662 residential properties across a sample of 35 unplanned low-income sub-wards selected for upgrading in each of Dar’s three Municipal Councils. The owner, or when absent, the longest tenant, was interviewed using a structured questionnaire and the sanitation facility was inspected. Sampling, selection and survey details have already been described [9]. In brief, using two-stage population proportional sampling, a spatially dispersed representative random sample of 2% of residential properties was selected across all 35 sampled sub-wards which together comprised a population of 339,000 in 2002 or approximately 20% of Dar’s estimated 2002 unplanned population (1,695,000; 70% of city population) [11]. This study focusses on the survey data measuring residents’ pit emptying and fecal sludge management behaviors and willingness to purchase the new hygienic manual de-sludging and removal pit emptying service. 

### 2.1. Background-Study Population, Sanitation Access and Emptying Options

Sampled properties were a mix of family-owned/occupied (51%), mixed landlord-tenant occupied (39%), and tenant-only (10%) properties. On average, 10 people (3.5 households) resided at each property; only 27% were occupied by one household. Nearly all properties had a sanitation facility (99.2%) consisting of traditional pit latrines (88%), ventilated improved pit latrines (8%), pour-flush latrines (2%), and drum/tyre pit latrines (1.5%). Less than 2% were connected to a septic tank or sewer. Facilities were often in poor condition: the pit/tank of over 40% was full or within 25 cm of full with sludge; only 5% had >1 meter of empty capacity. Mean monthly household income and expenditures were TSH 152,711 (U.S. $130) and 89,490 (U.S. $76), respectively. Monetary values are reported in 2008 TSH or 2008 U.S. $ (converted at TSH 1175 per U.S. $), unless noted. See Jenkins *et al.* [9] for more population and sanitation facility details.

Five pit-emptying methods in use across Dar’s unplanned areas were identified earlier [9]. Of these, vacuum tanker and Vacutug are considered here as ‘hygienic’ or safe services as both can effectively separate waste from human contact [7]. Both use a hygienic mechanized extraction and transport method and dispose of collected fecal sludge (FS) at municipally authorized treatment facilities in Dar. The other three methods are considered unhygienic: pit diversion (PD) involves slowly draining or flushing sludge into an adjacent temporary hole on the property after breaking open the side of the pit; flooding out (FO) involves intentionally releasing sludge into the neighborhood by unplugging a drain pipe installed in an elevated or exposed portion of the pit, often timed with heavy rains; manual emptying with a bucket (MB) is done by so-called ‘frogmen’ (*vyura*) who empty the whole pit or just the top portion, after breaking open the slab. Each involves direct contact with fecal waste during emptying and/or discharge of the sludge into the local environment. Only 34% of residential properties had access to a hygienic emptying service defined as having both plot physical access and local availability of either vacuum tanker or Vacutug services, while at 28% of properties, the facility’s pit was fitted with a drain pipe for flooding out [9].

In 2008, the international non-governmental organizations WaterAid and GOAL, together with researchers from the London School of Hygiene and Medicine, began work in Dar to design a new hygienic manual emptying service using the Gulper pump to extract pit sludge. At the time of the survey, a small feasibility test of the technology, equipment set-up, and operational parameters for service delivery showed promising technical and financial performance [12]. Testing indicated a viable target price of TSH 5,000 (U.S. $4.25) per 50 L drum of sludge removed (including dumping fee) with a minimum of four drums per service visit. These parameters were used to frame an assessment of willingness to pay for and purchase a hygienic pit emptying and removal service as described below. 

### 2.2. Survey Sample and Topics

Participants were the property owner (88%) or head of the longest tenant household (12%). Topics included perceptions of community problems and sanitation conditions; pit FSM practices; knowledge, attitudes, preference and past and future use of emptying methods/services and their costs; interest in and willingness to purchase the proposed emptying service; and socio-economic and other characteristics of respondent households and properties. Respondent households were categorized into income quintiles based on reported monthly income and those living below the Tanzania national basic needs poverty line (TSH 13,998/month in 2007) were identified [13]. Median monthly income ranged from U.S. $40 (quintile 1, poorest) to U.S. $255 (quintile 5, richest). Based on income, 17% lived below the poverty line, whilst 35% did so, based on reported expenditures. 

### 2.3. Willingness to Purchase the Gulper Service

Respondents were read a brief description of the proposed Gulper service and shown a photo from testing underway at the time. A series of questions was asked to assess: (i) level of interest and initial reactions, (ii) price perceptions (a cheap and expensive price bid), and (iii) willingness to purchase the service at TSH 5,000 per 50 L drum of sludge removed. Presentation materials and willingness to purchase questions are provided in the Supplemental Materials. 

### 2.4. Emptying Frequency and Costs

We calculated emptying frequency in years for each emptied latrine from the data on when the current facility was built, when it was last emptied, and total number of times it had been emptied. Reported expenses to empty the last time, including repair or replacement of a slab damaged during emptying, were adjusted for inflation to 2008 values and divided by the emptying frequency to estimate annual equivalent emptying costs. Equivalent costs per user household were estimated by dividing emptying costs by the reported number of households residing at the property who shared the facility. Emptying expenditure is compared to monthly income of respondent households and to the median monthly income of each income quintile, to assess ability to pay and affordability. While the number of households residing at each property and using the latrine was collected, we obtained socio-economic data for the respondent household only. As a result, in assessing affordability of per user household equivalent costs, we assume households residing at each property are in the same income quintile as the respondent household (*i.e.*, property owner, in most cases). 

### 2.5. Pit-Emptying Market Size

To estimate the annual number of facilities emptied and associated market value, emptying rates, mean amount spent to empty, and the density (population/facility) and types of sanitation systems from the survey were extrapolated to Dar’s estimated 2010 *unplanned* population (2,374,000) based on an annual growth rate of 4.3% [11]. 

### 2.6. Statistical Analyses

Descriptive and comparative analyses were used to examine perceptions of sanitation problems, fecal sludge pit management practices, and knowledge, attitudes and use of pit emptying methods. Associations were tested for significance using Chi-squared for proportions and ANOVA for group means. Logistic regression was used to calculate odds ratios (OR) of never having emptied the latrine, and when emptied, of using a hygienic *vs.* unhygienic method. A hierarchical approach to multivariate regression modeling [14] was used to examine factors explaining price bids and willingness to purchase the new hygienic emptying service at the offer price. Five blocks (groups) of factors were considered: 

Block 1: Household socio-economic and property occupancy characteristics

Block 2: Location-related factors

Block 3: Sanitation facility characteristics

Block 4: Pit emptying experience, knowledge, and attitudes, including initial attitudes towards the new emptying service offer 

Block 5: Pit fullness

Blocks of variables in the above order were added one block at a time and variables significant at the *p* < 0.05 level retained before moving to the next block. 

## 3. Results and Discussion

### 3.1. Existing Conditions

One in four surveyed residents (24%) cited poor household sanitation as a major problem facing their community, while over half (56%) cited poverty. Specifics of poor sanitation included flooded latrines, poor quality latrines, and lack of government involvement in latrines. Other major problems were poor drainage (40%, including no drainage, flooding, high water table, and mosquitoes), garbage (32%), lack of clean water (29%), and security (24%).

Only 13% considered household sanitation in their community to be good or very good; 65% considered it bad or very bad. Increasingly negative views were correlated with higher reported rates of frequent flooding during or even outside the rainy season *vs.* occasional, rare or never flooding (χ^2^ *p* < 0.001). Positive views were correlated with access to an improved sanitation technology as defined by the Joint Monitoring Program (χ^2^ *p* = 0.001), and with access to a hygienic emptying service, defined as plot access and local service availability (χ^2^ *p* = 0.024). When asked to identify the single biggest problem related to household sanitation, residents most often cited flooded latrines (24%), full latrines (20%), and lack of emptying services (14%). Also mentioned were poor security in latrines (11%), collapsing latrines (9%), and poor quality latrines (8%). Negative views were correlated with mentioning full latrines and flooded latrines as the biggest household sanitation problem, at 4.2 and 2.5 times, respectively, rates mentioned by those with a positive view (χ^2^ *p* < 0.001). 

### 3.2. Fecal Sludge Management Practices

We identified three common practices used to manage fecal sludge accumulation in sanitation facilities in study areas: pit additives, replacing the latrine, and emptying the pit. 

#### 3.2.1. Pit Additives

At over half of properties, users intentionally added a variety of products to the sludge pit to manage reported problems associated with on-site sanitation systems, the most common being bad odors followed by insects (see Supplemental Materials, Table S1: Products intentionally added to the fecal sludge pit of latrines in unplanned study areas of Dar Es Salaam). Residents with full latrines were more likely to report using an increasing number of products (*p* = 0.022). Addition of “salt”, ashes, old batteries, diesel, or paraffin in an attempt to reduce sludge volume, referred to as “sinking the sludge” (SS), was reported by 8.5% of owners at an average expenditure of $17 per treatment. 

#### 3.2.2. Latrine Replacement

Overall, 41% of sanitation facilities were a replacement for the original facility at the property. According to the decade when they were built, the proportion of latrines which were in use which were a replacement, ranged from 39% (in the 1990’s) to 52% (in the 1970’s). A full pit was the reason for replacing the original latrine in 43% of cases (17.6% of all facilities); other reasons for replacement were collapse or poor condition of the previous latrine (33%) and to have a more modern latrine (6%). 

#### 3.2.3. Pit-Emptying: Awareness, Availability and Methods Used 

Awareness and availability of the two hygienic methods and the three unhygienic methods for emptying sludge from latrines in Dar varied greatly (Table 1). 

**Table 1:** Knowledge, local availability, and use of fecal sludge pit latrine emptying or in-situ reduction methods across unplanned study areas of Dar Es Salaam.

Sample Subset:	Aware % (rank)	Preferred % (rank)	Available % (rank)	Used Last Time % (rank)	Use Next Time % (rank)
	All N = 662	Of those Aware	All N = 662	Emptied Latrines N = 241	Plan to Empty N = 360
**Hygienic methods:**					
Vacuum tanker	95 (1)	66 (1)	58 (2)	18 (2)	31 (2)
Vacutug	51	24 (2)	24	5	6
**Unhygienic methods:**					
Pit diversion (PD)	94 (2)	13 (3)	78 (1)	59 (1)	42 (1)
Manual bucket (MB)	77 (3)	11	56 (3)	5	16 (3)
—top of pit	68	5	47		7
—whole pit	33	6	23		9
Flood out (FO)	59	10	43	12 (3)	1
**In-situ methods:**					
“Sink sludge” (SS) *	28	5	18	2	3
**Summary of methods knowledge, availability and hygienic access:**					
Number of:		Mean	S.D.	Min–Max	Median
Methods known (includes FO, SS)		4.3	1.34	1–7	4
Methods known (excludes FO, SS)		3.4	1.04	1–5	3
Methods available (excludes FO, SS)		2.3	0.96	0–5	2
*Hygienic* methods available		0.8	0.71	0–2	1
Access to hygienic emptying service: (locally available + plot accessible) % (n)		34 (225)			
**Emptying rates **(properties were facility has been emptied):					
Overall (N = 660)		36%			
Original latrines (59%)		20%			
Replacement latrines (41%)		58%			
Replacements for a full latrine (16.5%)		75%			

* Pit additives (see Table S1, Supplemental Materials).

Overall, there was relatively widespread availability of pit diversion (PD: 78%) followed by tanker (58%), and manual bucket (MB: 56%). Over 40% reported that flooding out (FO) was locally available and nearly 60% knew the method. Availability of FO was positively associated with reporting lack of emptying services (χ^2^ *p* = 0.01) or poor quality latrines (χ^2^ *p* = 0.038) as the main community household sanitation problem and negatively associated with mentioning full pits (χ^2^ *p* = 0.15). 

Residents reported a median of two unhygienic methods and one hygienic method available in their community, although 35% had no hygienic method available. When accounting for vehicle accessibility to the plot, only 34% of properties had hygienic emptying service access (Table 1). Residents in one Municipal Council (Kinondoni) compared to the other two (Illala, Temeke) were less likely to report Vacutug service availability (χ^2^ *p* = 0.04) and more likely to report availability of FO (χ^2^ *p* = 0.005), MB whole pit (χ^2^ *p* = 0.007) and MB top of pit (χ^2^ *p* < 0.001). 

Sixty-four percent of property owners had yet to empty their sanitation facility. Among those that had, nearly half (44%) had emptied it multiple times. The three most common methods used were PD, vacuum tanker, and FO (Table 1). A hygienic method was chosen less than 25% of the time. Access to a hygienic emptying service increased the odds of using a safe emptying method by a factor of 23 and decreased the odds by 95% of using FO, the most environmentally dangerous method, compared with those lacking access, controlling for income (Table 2).

Income showed significant effects on choice of method. Households in the lowest income quintile (Q1) compared to the highest (Q5) were 85% less likely to use a hygienic method (OR = 0.15) and nearly four times more likely to use FO (OR = 3.8), independent of hygienic access (Table 2). 

**Table 2:** Odds ratios for emptying method as a function of access to a hygienic emptying service, adjusted for household income of residents who had emptied their facility (n = 241) in unplanned areas of Dar Es Salaam (2008).

Access to Hygienic Emptying Service (Exposure)	Method used to Empty (Outcome)			Income Quintile effect (Relative to Wealthiest Q5)		
	Method Used	Adj. OR	95% CI	Adj. Q1 OR	95% CIs	Income *p*-value
Model 1	Hygienic *	23.0	9.6–54.6	0.15	0.03–0.83	0.15
Model 2	Flooding out **	0.054	0.007–0.41	3.84	0.90–16.3	0.027

* Vacuum tanker or Vacutug service *vs.* unhygienic (pit diversion, manual bucket, flooding out) method; ** Over any other emptying method.

#### 3.2.4. Pit Emptying Frequency 

Emptied latrines were significantly older on average than never emptied latrines (18 *vs.* 12 years, *p* < 0.001), but the study also documented the existence of some very old latrines which had never been emptied. If original latrines that do not fill up are less likely to be replaced, this would explain existence of very old never emptied latrines. In fact, replacements were significantly more likely than original facilities to have been emptied (latrine age adjusted OR = 4.8; 95% CI: 3.3–7.1) and the odds of emptying a replacement built specifically to replace a full latrine were even higher (adjusted OR = 11.8; 95% CI: 6.7–21).

Among facilities that had been emptied, estimated emptying frequency in years for each of the main pit construction types in use (see Jenkins *et al.* [9] for shares of each type) was determined to be:

8.2 years (unlined)

6.5 years (partially lined)

8.5 years (fully lined)

4.7 years (drum/tire)

5.5 years (other, mainly septic and sewer)

Average emptying frequency decreased with each additional time a facility’s pit had been emptied, from 14.4 years for latrines emptied once, to 4.8 years for latrines emptied four or more times, confirming local perceptions that it takes less time to refill a latrine once emptied. We attribute this mainly to a failure of available methods to remove all of the contents of full pits, in particular solids that build up in the bottom of the pit, such that each successive emptying event results in diminished pit storage capacity. Other possible causes include on-going solid waste or sand accumulation in pits that is difficult to remove and structural damage to unlined or partially lined pits during empting.

Looking to the future, the majority of respondents (54%) had no idea when their facility would become full, while 20% expected it to fill within a year and the rest (26%) in more than a year. Overall, 60% planned to empty their facility when it became full, 15% intended to replace it, and the remainder (25%) did not know what they would do. The proportion of latrines whose owners plan to empty them (60%) is considerably higher than the portion of latrines emptied to date (36%) pointing to an increasing trend of emptying rather than replacing full latrines. Of those intending to empty, 81% said it was the owner household’s responsibility alone to decide and manage the emptying. Shared decision making responsibility by users was reported at just 16% of properties.

#### 3.2.5. Pit Emptying Costs and Affordability

Median and average expenditure to empty including slab repair, a frequent cost, was U.S. $35 and U.S. $57, respectively. Mean expenditure varied by method, from a low of U.S. $40 for FO to a high of U.S. $65 for Vacutug, although cost differences were not significant given limited sub-samples (see Supplemental Materials, Table S2: Amounts paid to empty sanitation facilities in unplanned areas of Dar Es Salaam). Annual equivalent cost per user household ranged from $1.50 (Vacutug) to $6.50 (MB). Annual equivalent cost per individual user (adults and children ≥ 3 years) ranged from U.S. $0.68 for PD to U.S. $1.35 for MB. 

Annual emptying cost per user household amounted to less than 0.5% of annual household income on average, assuming user households at each property have an income similar to the respondent household (Table S2). While costs per year represent a small and quite affordable portion of income, median cash expenditure to empty (U.S. $35) represented nearly 100% of the median monthly income of the poorest 20% of respondent households and a third of the median monthly income (U.S. $106) across all respondent households. When responsibility falls on the owner’s household as indicated here, having enough cash available to pay for emptying when the need arises may pose a serious constraint, and for the poorest owners, an insurmountable barrier to paying for emptying. Even when divided among user households, median emptying expenditure of U.S. $4.30 per user household represents more than 10% of the median monthly income of the lowest income quintile (poorest 20%) of households in the study population (as represented by the income distribution of respondent households), and 36% of the Tanzania basic needs monthly income, below which 17% of study respondent households’ income fell. Of those planning to empty their latrine when full, just 34% would not require time to save to pay for the service; 14% would require up to 3 months, while 23% would require more than 3 months. Almost a third, despite intending to empty in the future, had no idea what it would cost and if they would need to save. Reported saving times are consistent with the range of estimated months required to save if a property owner household were to put aside 5% of their monthly income to save for emptying, assuming the average income of each income quintile across respondent households (see Supplemental Materials, Figure S2: Time required to save for capital costs of sanitation facilities and pay for safe pit emptying service at 5% of average quintile household income in unplanned areas of Dar Es Salaam).

### 3.3. Preferences, Perceptions and Choice of Emptying Method

Among those aware of each method, tanker service had the highest rate of preference at 66%, followed by Vacutug (24%) (Table 1). Despite a clear stated preference for one of the hygienic methods (75% overall; 71% of those who had emptied before), only 25% actually used one. 

**Figure 1:**
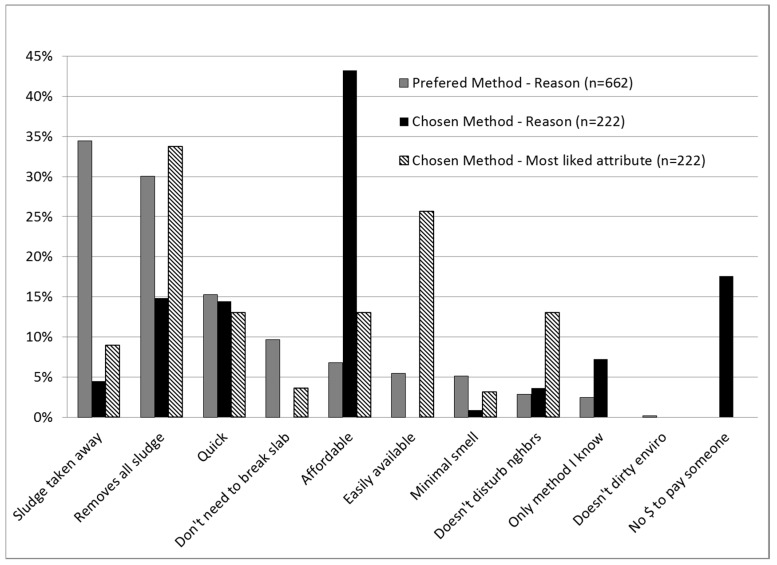
Positive perceptions shaping preference for latrine emptying method *vs.* reasons for choosing the method used last time in unplanned areas of Dar Es Salaam (2008).

**Figure 2:**
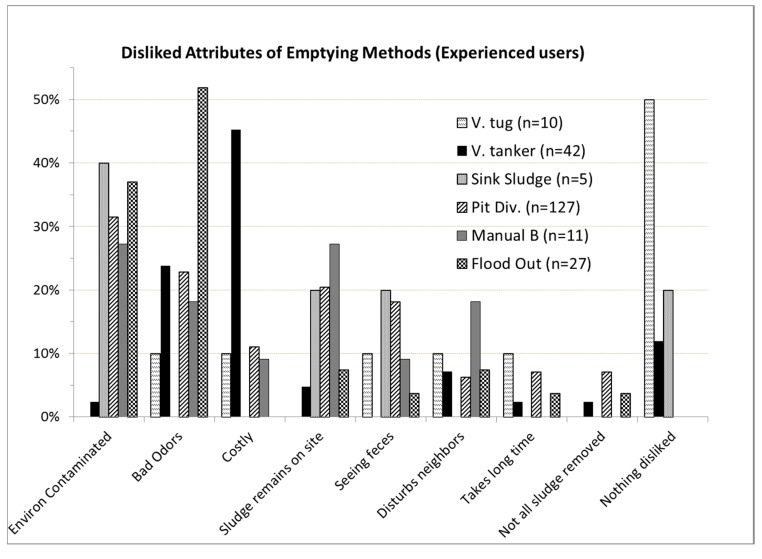
Negative perceptions of the emptying method used last time to empty the latrine facility in unplanned study areas of Dar Es Salaam (2008).

Positive and negative perceptions of existing emptying methods and the reasons for choosing the method used last time are shown in Figure 1 and Figure 2. Two attributes dominated method preference: “sludge taken away” and “removes all the sludge”. These however, were different from the factors that determined choice of method to ultimately use when need arose, namely, affordability, and “no money to pay someone” (Figure 1). Beyond affordability and lack of cash, easy availability and ability to remove all the sludge appear to be important characteristics influencing choice of method according to what users liked most about the method they chose (Figure 1). Negative perceptions of unhygienic methods (Figure 2) included contamination of the environment (32% of users), bad odors (26%), and that the sludge remained on site (19%). Hygienic methods were mainly perceived negatively for their high cost (38%), although they were also sometimes negatively viewed for causing bad odors (21% of users) and disturbing the neighbors (8%). Tanker service was more negatively perceived than Vacutug, both for high cost (45% *vs.* 10%) and bad odors (24% *vs.* 10%).

### 3.4. Estimated Market for Emptying Services

During the 12 months preceding the survey, at 8.3% of surveyed residences the sanitation facility had been emptied. Assuming sanitation conditions in sampled sub-wards were generally representative of Dar’s unplanned areas, approximately 235,000 latrines and 2300 septic tanks served Dar’s unplanned population in 2010. Applying the observed emptying rate, about 20,000 latrines and septic tanks were emptied in 2010, representing over U.S. $ one million in annual revenue for informal and formal service providers. Three quarters (15,000) would have been emptied using an unhygienic method with the sludge released in the neighborhood to contaminate the environment with fecal pathogens. An estimated 2400 of these would have been emptied by actively flooding out a portion of the pit (*i.e.*, unplugging the drain pipe). 

### 3.5. Demand for the Proposed New Emptying Service 

Reactions to the proposed new pit emptying service, respondents’ perceived “cheap” and “expensive” price bids, and willingness to purchase (WTP) amounts at the service offer price are summarized below and findings from the hierarchical regression models (presented in Table 3) of the factors associated with price bids and WTP are reviewed.

#### 3.5.1. Perceptions of the New Service 

**Figure 3:**
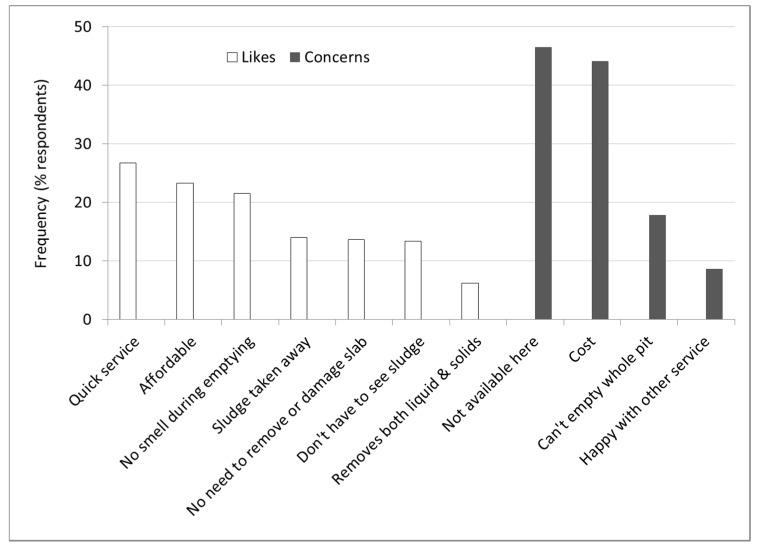
Positive and negative reactions to the proposed Gulper pit emptying service offer in unplanned study areas of Dar Es Salaam (2008).

Respondents were highly interested in the new service (77% “very interested”; only 1.1% neutral or uninterested). Most found it “very new” (76%) and “very different” (78%) compared to services and methods they knew. Reactions to the service suggest that quickness, affordability, and no smell were its most attractive features (Figure 3). Four concerns were expressed of which service cost and availability were each shared by over 40% of respondents (Figure 3). 

Female property owners reacted with greater interest than males (82% *vs.* 72% “very interested”; *p* = 0.005) to the presentation of the service and were more attracted than men to lack of smell during emptying (24% *vs.* 18%; *p* = 0.07) and sludge taken away (17% *vs*. 10.5%; *p* = 0.013), but otherwise responded similarly. Households with incomes in the bottom 40% compared to the top 60% were less likely to perceive the Gulper service as “very new” (71% *vs.* 81%; *p* = 0.023) or “very different” (71% *vs*. 85%; *p* < 0.001) from services they knew, and less likely to be “very interested” (68% *vs.* 85%; *p* < 0.001). They were more likely to mention not having to see the sludge during emptying as an attractive feature (18% *vs.* 11%; *p* = 0.018) and less likely to mention cost as a concern (31% *vs.* 52%; *p* < 0.001), but otherwise reacted similarly. 

#### 3.5.2. Service Price Bids

**Figure 4:**
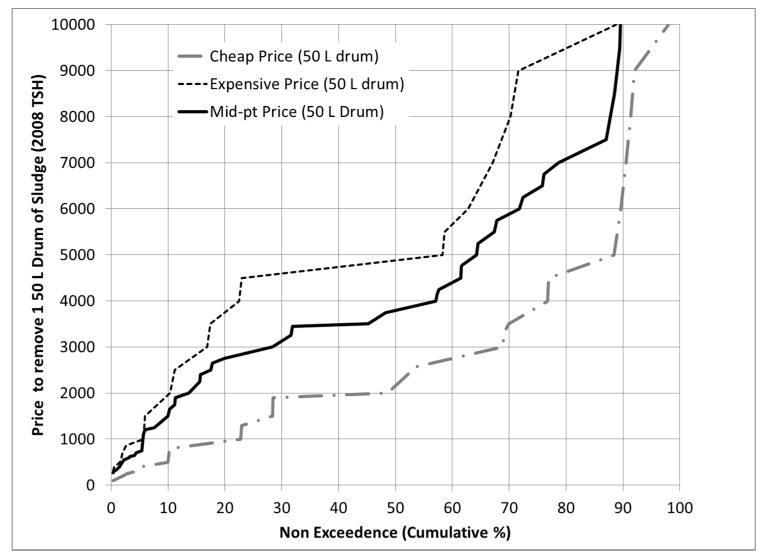
Proposed Gulper service “cheap” and “expensive” price bids for removing one 50 liter drum of fecal sludge from the latrine pit and property. Non-exceedance % is the fraction of respondents who named a price equal to or less than the Y-axis price. The mid-point price is the respondent’s average bid (cheap and expensive price average).

Cumulative distributions of respondents’ perceived “cheap” and “expensive” price for removing 50 liters of sludge are shown in Figure 4. The median cheap and expensive price was TSH 2200 (U.S. $1.87) and TSH 4800 (U.S. $4.09), respectively. The service offer price (TSH 5000/drum) exceeded the cheap and expensive prices of 56% of respondents. Factors significantly associated with the likelihood of expressing an expensive price below TSH 5000/drum (Table 3, model 1) related to location (Block 2, 63% of model R^2^); pit emptying experience, knowledge and attitudes (Block 4, 32%), and to a lesser extent, household and property characteristics (Block 1, 5%). No latrine (Block 3) or pit fullness (Block 5) factors were significant.

Significant location factors indicate that respondents in Kinondoni Municipal Council (MC) (*p* = 0.009) or in areas where FO was locally available (*p* = 0.005) were more likely to state an expensive price below the TSH 5000 target price. On the other hand, respondents with plot vehicle access (*p* = 0.002) or in high water table areas (*p* = 0.017) were more likely to name an expensive price above TSH 5000. Three factors related to emptying experience, knowledge, and attitudes were significant: being concerned about the new service cost (*p* = 0.019), that it cannot empty the whole pit (*p* < 0.001), and having more emptying services locally available (*p* = 0.011) each increased the probability of an expensive price below the TSH 5000 target. Finally, households with expenditures below basic needs had significantly increased odds of an expensive price below TSH 5000 (*p* = 0.004). 

#### 3.5.3. Willingness to Purchase (WTP) the New Service

At TSH 5000/drum, only 4% of respondents expressed unwillingness to purchase any amount of the offered service. Among the 96% WTP, median and average number of drums was four (TSH 20,000) and five (TSH 25,000), respectively. Overall (N = 662), 57% were WTP ≥ 4 drums (the target amount), of which 42% were ready to pay “now” (24% overall). Among those ready to purchase ≥ 4 drums “now”, the median amount was six drums (TSH 30,000). Awareness of Vacutug, MB top of pit, and FO emptying methods were associated with higher WTP amounts (*p* = 0.074, 0.023, and 0.05, respectively), while awareness of tanker services and MB whole pit were associated with lower WTP amounts (*p* = 0.02 and *p* = 0.013).

In multivariable modeling (Table 3, model 2), emptying experience, knowledge and attitude related factors (Block 4) accounted for over half of predicted WTP four drums or more. WTP ≥ 4 drums was positively associated with liking the new service because it took sludge away (*p* = 0.001) or because it had no smell (*p* = 0.022), absence of concerns about its cost (*p* = 0.05), a perceived expensive price above the offer price (*p* < 0.001), a perceived cheap price above the offer price (*p* = 0.011), not knowing any hygienic emptying methods (*p* ≤ 0.02), or having none locally available (*p* = 0.038). Those who had emptied their latrine using the only available option (no options to choose from) (*p* = 0.041) or whose reason for the method used was to empty the whole pit (*p* = 0.051), were also more likely to be WTP ≥ four drums. 

**Table 3:** Hierarchical logistic regression models of price perception and willingness to purchase the proposed Gulper service at TSH 5000/50 L drum.

Block	Variables	Levels	N	TSH 5K > Expensive price 56% (n = 301/535)			WTP ≥ 4 drums ^a^ TSH 5K 57% (n = 305/535)			Ready to pay NOW38% (n = 117/305) ^a^		
				Model 1			Model 2			Model 3		
				ßeta	*p*-value ^b^	Exp(ß)	ßeta	*p*-value	Exp(ß)	ßeta	*p*-value	Exp(ß)
	Constant		535	−1.36	0.002	0.26	4.24	<0.001	69.3	-3.08	0.001	0.046
**Block 1:** Household & property characteristics	Expenditures/month < basic needs	Yes (No = ref)	199	0.589	0.004	1.80	−080	<0.001	0.45			
	Housing type	Tenant-only (ref)	56				-	0.008	-	-	0.019	-
		Family only	267				−0.20	0.565	0.82	1.62	0.007	5.07
		Landlord-tenant mix	212				0.53	0.14	1.70	1.58	0.007	4.84
	Education	Uneducated (ref)	18				-	0.042	-	-	0.01	-
		Stand. 2–6	36				−1.40	0.117	0.25	−1.93	0.03	0.15
		Stand. 7	278				−2.13	0.009	0.12	−0.54	0.421	0.58
		Secondary completed	167				−1.84	0.026	0.16	−0.67	0.332	0.51
		Above secondary	36				−2.13	0.021	0.12	1.00	0.242	2.73
	Multi-household residence	Yes (No = ref)	387							1.68	<0.001	5.35
		**Block 1 Δ Nag.^c^ R^2^ :**			**0.012**			**0.11**			**0.17**	
**Block 2:** Location-Related	Municipality	Temeke MC (ref)	173	-	<0.001	-						
		Illala	175	−0.261	0.44	0.77						
		Kinondoni	187	1.031	0.009	2.80						
	Flooding out available in area	Yes (No = ref)	232	0.56	0.005	1.75				−0.335	0.271	0.72
	Vehicle accessible plot	Yes (No = ref)	208	−0.633	0.002	0.53						
	High water table	Yes (No = ref)	317	−0.798	0.017	0.45						
	Elevation (meters **^d^**)	Range: 7 to 45	−all−	0.02	0.097	1.02						
	Low elevation (< 20 m)	Yes (No = ref)	67							1.41	0.003	4.11
		**Block 2 Δ Nag.^c^ R^2^ :**			**0.14**			**0**			**0.057**	
**Block 3:** Latrine-	Flood out pipe observed	Yes (No = ref)	147				0.43	0.082	1.54			
	Below ground unlined	Yes (No = ref)	183							0.898	0.003	2.45
Related	Replacement latrine	Yes (No = ref)	225							−0.504	0.093	0.60
		**Block 3 Δ Nag.^c^ R^2^ :**			**0**			**0.015**			**0.057**	
**Block 4:** Emptying experience, knowledge & Attitudes	Sludge taken away (Gulper-Like most)	Yes (No = ref)	78				1.01	0.001	2.75			
	No smell (Gulper-Like most)	Yes (No = ref)	115				0.59	0.022	1.81			
	Removes liquid & solids (Gulper-Like most)	Yes (No = ref)	39							−1.703	0.015	0.18
	Cost (Gulper-Concern)	Yes (No = ref)	229	0.46	0.019	1.58	−0.42	0.05	0.60	−0.645	0.042	0.53
	Can’t empty whole pit (Gulper-Concern)	Yes (No = ref)	94	1.304	<0.001	3.68						
	Tsh5000 > Gulper expensive price	Yes (No = ref)	301	−ni−			−1.12	<0.001	0.30			
	Tsh5000 < Gulper cheap price	Yes (No = ref)	61	−ni−			1.38	0.011	3.96			
	Whole pit emptied (past choice reason)	Yes (No = ref)	29				0.94	0.051	2.56			
	No other service avail. (past choice reason)	Yes (No = ref)	11				1.81	0.041	6.09	3.62	<0.001	37.17
	Number of empty services available in area ^e^	Range: 0 to 5	-all-	0.27	0.011	1.31						
	Number of hyg. empty methods known	0 (ref)	24				-	0.056	-			
		1	250				−1.39	0.018	0.25			
		2	261				−1.38	0.02	0.25			
	Number of hyg. empty methods available in area	0 (ref)	195				-	0.038	-			
		1	247				−0.44	0.068	0.65			
		2	93				0.35	0.317	1.43			
		**Block 4 Δ in Nag.^c^ R^2^ :**			**0.07**			**0.22**			**0.08**	
**Block 5:**	Pit full or within 25 cm of full	Yes (No = ref)	153							1.161	<0.001	3.19
Pit condition		**Block 5 Δ Nag.^c^ R^2^ :**			**0**			**0**			**0.045**	
		**Model Overall Nag.^c^ R^2^ :**			**0.22**			**0.34**			**0.41**	

**^a^** Model 3 examines factors associated with being ready to purchase now, among the subset of respondents who were WTP ≥ 4 drums (n = 305); **^b^** Wald p-value; **^c^** Nagelkerke R**^2^**; **^d^** Meters above sea level; **^e^** Includes vacuum tanker, Vacutug, pit diversion, manual bucket top only, and manual bucket whole pit, but excludes flooding out and sinking the sludge; NB. Tested factors not significant in: Block 1: respondent gender, household income in bottom 40% (*vs.* top 60%); Block 2: frequent flooding, availability of hygienic service; Block 3: improved technology; Block 4: method used last time to empty if emptied, other stated Gulper likes/concerns (see Figure 3), number of times emptied; Block 5: years since last emptied.

Household and property characteristics (Block 1) explained the next biggest portion (32%) of overall model R^2^. Mixed landlord-tenant (*vs.* tenant- or family-only properties) expressed higher WTP amounts (*p* = 0.14), at or above the 4 drum minimum. Household expenditures below basic needs (*p* < 0.001) and higher levels of education (*p* = 0.042) were associated with lower WTP amounts, below four drums. One latrine-related factor (Block 3), the presence of a flood out drain pipe, was positively associated with higher probability of WTP ≥ 4 drums (*p* = 0.082). No location or pit fullness factors (Blocks 2 or 5) met the inclusion criteria. 

#### 3.5.4. Ready to Purchase and Pay Now 

Among those WTP ≥ 4 drums (n = 305), factors from all five blocks explained differences in being ready to pay now (Table 3, model 3). Ready to purchase requires cash on hand or the ability to mobilize it quickly. It would also indicate a pressing need or desire to empty or reduce pit volume, as well as confidence in and an absence of concerns about the proposed service. We found that household and property characteristics (Block 1) contributed the greatest portion of explanatory power. Socio-economic and occupancy factors positively associated with ready to purchase now were family-only and landlord-tenant occupancy (*vs.* tenant-only) (*p* = 0.007), multi-household residence (*vs.* single household) (*p* < 0.001), and level of education (*p* = 0.01). Emptying experience, knowledge and attitude factors (Block 4) provided the next biggest contribution. Those who had emptied their latrine before using the only available option (*p* < 0.001) or who did not express a cost concern for the new service (*p* = 0.042) were more likely to be ready to pay now. Those attracted to the new service because it could remove liquid and solids were less likely to be ready to pay (*p* = 0.015).

Several location-, latrine- and pit fullness-related factors also predicted whether a person WTP the target amount was ready to pay now. Living in an area where FO was locally available reduced the likelihood of being ready to pay (*p* = 0.27), while low elevation location (< 20 m) increased it (*p* = 0.003). Having an unlined pit (*vs.* partially or fully lined) (*p* = 0.003), an original (*vs.* replacement) latrine (*p* = 0.093), or a pit that was nearly or completely full (*p* < 0.001), each increased the likelihood of being ready to pay now.

## 4. Discussion

We applied a consumer sanitation demand behavioral approach (e.g., [15]) to examine current fecal sludge management (FSM) problems and behaviors of households and to understand demand for improved FSM services in Dar’s unplanned settlements where on-plot sanitation, predominantly pit latrines, are used by >99% of the population.

### 4.1. Characteristics of the Problem

Despite near universal access to and use of a toilet facility, the majority of households perceived sanitation conditions to be very poor and were regularly exposed to discharges of untreated fecal waste from household facilities within their communities. Risk of exposure to fecal sludge (FS) was associated with environmental vulnerability, lack of access to hygienic emptying services, and poverty. 57% of residents reported problems related to full pits (full pits, flooding of full pits, and lack of emptying services) as the single biggest sanitation problem in their community. Residents’ perceptions were consistent with finding 40% of sludge pits to be completely or nearly full, and two thirds of residential properties without access to a hygienic emptying service.

Pit latrines are a sanitation technology designed for low-density settings where space to dig a new pit and build a new latrine will be available when the current facility’s pit fills. Approximately 70% of Dar’s population live in unplanned and densifying settlements where digging a new pit is increasingly constrained by lack of space [3]. In study sub-wards, gross density in 2002 averaged 379 people/ha and ranged as high as 631. A further constraint to replacement was the average cost of a basic pit latrine (U.S. $272) [9] relative to low incomes (study population median of U.S. $106/month) and high poverty rates (17% to 35%) in the unplanned study areas. Consequently, simple replacement, let alone upgrading to a more hygienic (expensive) water-seal technology or fully lined pit to facilitate mechanical emptying, is likely to be unaffordable for many residents and significantly raises annualized capital costs of providing for basic sanitation. 

While the data demonstrate that latrine replacement has been, and will continue to be a choice of some households in Dar, full latrines are increasingly being emptied rather than replaced. The high prevalence of unsafe emptying—over three quarters of emptying events—combined with increasing rates of emptying, point to access to safe pit-emptying services being at least as important as access to sanitation facilities to protect the population from exposure to fecal pathogens. 

The high rate of unhygienic emptying occurred despite over 70% of residents preferring a hygienic service for the main reason that it removed sludge from the area. Unhygienic methods in Dar leave sludge on the property or release it into the neighborhood. Users disliked these methods because they contaminated the environment and caused bad odors, revealing a degree of awareness of their dangers and consumer dissatisfaction with using them. Key barriers to hygienic emptying, despite widespread preference, were lack of availability and access, compounded by high cost relative to income. Households with accessible plots and service availability were on average 23 times more likely to use a hygienic service than those without such access, adjusting for income, while poverty had an added effect such that the lowest compared to the highest income quintile were 85% less likely to empty hygienically, despite access. We note that average tanker service costs in Dar (Table S2) are in line with costs and prices reported for mechanical extraction and haulage services in other cities in Sub-Saharan Africa and Asia, irrespective of whether sludge was treated [16,17,18,19,20]. 

Availability in the community and ease of obtaining services were key factors determining choice of which emptying method to use in Bamako, Mali, and two cities in Bangladesh [17,21]. In Bangladesh, NGO-operated hygienic Vacutug services were subsidized and affordably priced at the same rate as manual emptying, but were difficult to obtain and arrange, and unavailable on weekends and evenings when owners were home to supervise the work, resulting in very low uptake [21]. In Bhutan, lack of awareness and knowledge of how to obtain pre-paid municipal empting services was a key barrier to their use by urban septic tank owners, while in both Bhutan and the Philippines, lack of understanding of the need for regular de-sludging or how to determine when de-sludging was needed were further barriers to timely hygienic emptying [22,23]. 

In Dar we also found that many households, up to 30%, preferred unhygienic methods because these methods removed all the FS from the pit and were more affordable than existing hygienic services. Dissatisfaction with vacuum tankers has been reported by urban pit latrine owners for their inability to empty the pit completely, lack of price transparency, and unpredictably high costs of service compared to manual emptiers [17]. Large mechanical suction tankers are often an inappropriate and poorly matched technology for emptying pit latrines. Vacuum tanker suction risks damaging unlined pit walls, tank capacities are oversized relative to pit volumes, and suctioning more concentrated pit latrine fecal sludge (compared to septic tanks) which may contain some fraction of solid waste, is often difficult if not impossible without addition of water to the pit to dilute the sludge, something tanker businesses are reluctant or unwilling to do because of the added time and expense [7,16,24,25]. 

With little or no spare cash to pay and hygienic services both expensive and difficult or impossible to obtain or use, some households in Dar’s unplanned areas have resorted to flooding out (FO), the most dangerous method observed. Where there was access to hygienic services, FO was less prevalent, but the poorest households, independent of access, were still more likely to use FO than wealthier ones. The practice, where physically feasible, unlike other methods, requires no cash once the drain pipe has been installed. We found evidence of broad awareness and local exposure to this highly unsafe practice (43% said it was practiced in their community). An analysis of latrine construction by decade built showed the rate of installation of a flood out drain pipe has been increasing and in some sub-wards rates are much higher than in others [9], suggesting growing use and concentration of the practice in certain parts of the city.

Another emerging consequence in Dar of very limited physical and economic access to tanker and Vacutug emptying services, and no affordable, appropriate, and safe alternatives, is far too infrequent emptying of latrines. Average emptying frequency was every 7.5 years for latrines that had been emptied while 48% of never emptied latrines were over 10 years old. These rates are well below average frequencies of empting pit latrines or de-sludging septic tanks in poor urban neighborhoods elsewhere, for example in Bamako (1.7 years; <10% of latrines over 10 years old had never been emptied), Ougadougou (6 to 12 months), and Nouakchott (7 months), or recommended for latrines in South Africa (<5 years) and sludge tanks in Bhutan (2 years) and the Philippines (3 years), [17,19,23,25,26]. 

Considering that long-term FS accumulation rates in pit latrines may range from 10 L/p/year (dry, well drained) to 100 L/p/year (wet, poorly drained) with an average of 25 L/p/year [16], a typical facility in Dar (10 users), could accumulate 250 L/year and as much as 1000 L/year of FS, excluding contributions of solid waste disposed into the pit. A latrine with 2.5 m^3^ pit storage capacity (e.g., 1 m diameter by 3 m deep) would thus be full within 10 years, on average, and in as little as 2.5 years, where pit conditions are wet and water logged, as they are likely to be in frequently flooded and high water table areas of Dar, or where water is added to pits, a practice widely used in Dar [9]. Too infrequent emptying in Dar’s unplanned areas is supported by our finding of over 40% of latrines’ pits with less than 25 cm of empty space below the slab and 8% completely full, translating to as many as 100,000 latrines with dangerously full pits across Dar’s unplanned areas in 2010. Consequences of overly full pits include FS regularly overflowing from facilities into residences, neighborhoods and water ways during the rainy season from flooding and high water tables; highly unsanitary and unpleasant conditions in latrines which increase the risk of direct contact with FS during use, especially for children; increased exposure to flies, insects and vector-associated disease transmission, and increased contamination of shallow groundwater. 

The picture that emerges for the urban poor in Dar is of expensive, difficult to obtain, inappropriate, and unsafe sanitation service choices. Households delay emptying as long as possible, continuing to use full pits well beyond what can be considered hygienically safe while coping with the immediate negative effects by purchasing a host of pit additives, some or many of which are unlikely to be effective. Eventually, they face spending considerable amounts for mostly unhygienic emptying, or resorting to the dangerous practice of flooding out sludge from the upper portion of their pit. 

### 4.2. Demand for Improved Emptying Services 

Given this situation, near universal interest in the proposed Gulper service and willingness to pay something for it (96% indicated at least TSH 5000 (U.S. $4.30)) is unsurprising and may reflect the lack of alternatives and desperation in which residents find themselves. Better evidence of market demand for an affordable appropriate hygienic emptying service for pit latrines in Dar is the 57% of respondents willing to pay at least TSH 20,000 (U.S. $17) and a median of TSH 30,000 (U.S. $26) for removing 300 L of FS and the 24%, overall, who were ready to buy the service now. 

Strong interest and willingness to purchase the new service indicate a large latent demand for an improved pit emptying service in the community that is affordable, quick, and clean, does not smell, and takes the sludge away from the property. Low ability to pay and concerns about service cost, however, reduced the amount poorer households were willing to pay. More than half of those WTP the target minimum of 200 L were not ready to purchase because of time needed to save or preferring to wait until their the pit was completely full. 

If safe emptying services must be paid for in full at the time of service by the property owner, many property owners in Dar’s unplanned areas are either too poor or lack the cash on hand to purchase hygienic services, whether they be vacuum tanker, Vacutug, or the proposed new Gulper manual emptying service. They will be even less able to pay at the higher emptying frequencies needed to prevent exposure to fecal sludge from overly full pits. Delayed emptying because of the high one-time payment cost appears to prevail in other cities where large segments of the urban poor depend on on-site sanitation systems [22,23].

### 4.3. Recommendations towards Improving FSM for On-Site Systems

This research has identified both a clear need and consumer demand for an appropriate hygienic emptying service for existing pit latrines and unplanned settlement access conditions in Dar. Assuming viable business models for hygienic manual pit emptying using suitable small-scale pit emptying technologies (see Thye *et al.* [7] for a critical review of the Gulper and other technologies) can be operated and supported through provision of decentralized sludge discharge points under a municipal framework for FS collection, treatment and disposal [4,27], several demand-side and financing recommendations for improving FSM emerge from this research. 

#### 4.3.1. Cross-Subsidies for the Poorest Households 

The poorest households who cannot afford emptying even if costs are spread over multiple small payments will require subsidies. While examples of reverse (perverse) financial transfers for sanitation from poorer to better off households have been reported, for example, in Dakar where the large portion of residents without sewer access pays a water bill surcharge to subsidize operation of the sewer system which serves only a small portion of better-off households [20], there are few examples of cross-subsidies from surcharges on sewer bills or explicit use of tax transfers to subsidize safe FS emptying for the non-sewered urban poor, apart from South Africa [28]. Cross-subsidies for the poorest could be financed with a tax on sewer or water bills, on sanitation facilities, or property [29,30], and distributed to qualified poor households as targeted vouchers, cash rebates, or via other market-compatible subsidy mechanisms [31]. Alternatively, output-based subsidies could be paid to emptying businesses who service the facilities of qualified poor households or to certified emptying businesses for servicing every latrine in poor sub-wards when they discharge the FS at a local discharge point set up for each area [28,29,32].

#### 4.3.2. Financing and Incentives for More Frequent and Regular Safe Emptying of Pit Latrines

Financing mechanisms, incentives and regulatory changes for safe pit emptying are needed to address household cash flow constraints and induce more frequent and regular emptying. These should allow for households to make small affordable, more frequent and predictable payments or pre-payments for regular and even preventative emptying rather than the current situation where households face an unpredictable and often unaffordable payment after their facility has become dangerously full.

Assuming the average residential sanitation facility in study areas accumulates 250 L/year of FS plus up to 50 L/year of solid waste and the Gulper or similar viable pit latrine empting service were employed to remove 300 L of FS/year and discharge it to a nearby municipally controlled discharge point at a price of U.S. $26, this would represent a cost to property owners about U.S. $2.20/month (2008 value). If charged as a city property or sanitation facility fee, this amount could be affordable at ≤2% of monthly income for 47% of owner households (paying alone) or for 78% of user households (sharing costs). Major institutional changes in municipal oversight and responsibility for assuring safe on-site FS emptying would be required to establish systems for regular payment and contracting for annual or semi-annual emptying of pits, but systematic preemptive emptying of multiple facilities at one time in each neighborhood by service contractors has been shown to increase the cost-efficiency of delivering services while simultaneously creating safer environments [19]. A similar approach has been used to pre-finance and cross-subsidize regular preemptive contracted emptying of on-site sanitation systems every 5 years in Marikina City (Philippines) to ensure safe FSM for non-sewered low-income urban residents [33]. The increased and more predictable collected volumes of FS requiring disposal that would result from contracted regular pit emptying present another potential opportunity to further reduce emptying service costs through development of FS resource recovery oriented treatment [34,35]. 

#### 4.3.3. Standardized “Empty-Able” Pit Latrine Specifications and Construction Regulations 

Basic pit latrine models are rarely if ever designed or constructed with any view to providing for the necessary site access and access to the inside of the pit to be able to safely extract and remove fecal sludge from the premises for safe discharge at authorized FSM sites. This study found that many surveyed properties lacked direct plot access to hygienic emptying vehicles, and that latrine owners frequently incurred additional expenditure to repair or replace a damaged or destroyed slab floor during emptying because there was no other way to access the pit contents. In Dar, pit latrine design and plot construction modifications are needed to allow for easier physical access to the latrine site and to the inside of pits for cost-efficient and hygienic emptying, without having to destroy the pit slab or superstructure. Significant ongoing demand for latrine replacement and new latrines to accommodate high population growth rates in unplanned areas of Dar [9] present an opportunity to intervene and incentivize modifications to design and construction using rebates and regulations to ensure pit latrines can be easily and safely emptied by a locally available hygienic service suited to the settlement area. Reduced costs of construction could even be achieved through smaller sized pits, if regular hygienic pit emptying were mandated, operationalized, provided for financially, and institutionalized through a city- or area-wide safe FSM program. In the longer-term, research into consumer appealing innovative latrine technologies, including effective pit additives, is needed to develop better on-site sanitation solutions that reduce fecal sludge filling rates, and make emptying simpler and safer, and/or less frequently needed in Dar’s unplanned areas. 

## 5. Conclusions 

Whilst access and coverage are usually seen in terms of usage of a functional latrine, the bigger challenge in low-income dense urban settlements is likely to be physical and economic access to safe services for emptying—this is when fecal waste enters the environment causing public health problems. As such, in the post-2015 Sustainable Development Goals and low/middle income country urban planning goals, no sanitation policy should overlook this. This study has shown that most residents in Dar’s unplanned settlements use unhygienic methods to empty their latrines and do not empty pits as often or as thoroughly as they should, partly to save money or because they cannot afford better and partly because hygienic services are unavailable to them. As a result, populations are regularly exposed to fecal sludge in overly full pits during use and from frequent releases into local communities. Appropriately scaled hygienic emptying and haulage services based on operationally viable manual or mechanical pump technologies that can extract sludge from existing latrine pits in dense unplanned communities and move it from the plot to a controlled discharge point are part of the solution to the current problem. A large latent demand for such services was measured in Dar, including willingness to pay varying amounts up to and beyond the estimated small-business costs of delivering services by over 50% of property owners. However, without sustainable financing mechanisms to subsidize the service for the poorest owners, and make payment more affordable by spreading costs over multiple frequent payments for most others, and eventually regulatory efforts to promote the use of safe services and regular emptying, demand and uptake of services is unlikely to materialize at the scale and frequency needed to resolve FSM problems in Dar through the market alone.

## Supplementary Files

Supplementary File 1
