# Supplementary material for: Pit Latrine Emptying Behavior and Demand for Sanitation Services in Dar Es Salaam, Tanzania
Source: Int J Environ Res Public Health. 2015 Feb 27;12(3):2588–611. doi: 10.3390/ijerph120302588 (PMC4377920; doi:10.3390/ijerph120302588)
Supplement: Supplementary File 1 [file ijerph-12-02588-s001.pdf]

Supplementary Information

Pit Latrine Emptying Behavior and Demand for Sanitation Services in Dar Es Salaam, Tanzania

---

Manuscript Supplemental Material

Gulper Emptying Service Concept and Willingness-to-Purchase Related Questions

The following English translation of the Kiswahili verbal presentation (Box 1) and accompanying picture (Figure S1) were presented to respondents:

"I would like to introduce a potential new latrine emptying service that might be of interest to you. It is currently known as the "Gulper Service" ("*Huduma ya Gulpa*").This is a new latrine emptying service that is being tried in Azimio [sub-ward]. It has the following characteristics.

- It is affordable. You can pay for any quantity (in drums) of sludge that you would like to be removed.
- It removes both the liquids and the solids.
- It takes only a short time to extract the sludge.
- There is no smell during latrine emptying.
- One does not get to see or touch the sludge when it is being extracted.
- The sludge is taken away after being extracted from the pit.
- It accesses the pit contents without demolishing the slab or superstructure. This means there are no costs for re-building or repairing the slab."

The following questions were asked to assess interest in and willingness to purchase the Gulper service:

1. How interested would you say you are in this service? (choice of 5 ordered responses:: 1 = very interested, to 5 = not interested at all)
2. How new do you feel this service is to you? (1 = very new, to 5 = not new at all)
3. How different is it compared to other emptying services/methods that you are aware of? (1 = very different, to 5 = not different at all)
4. What would you say you like the most about the service? (open ended, one response)
5. What concerns would you have about the service? (open ended, multiple responses)
6. How much money would be cheap to remove one 50 L drum of pit contents?
7. How much money would be expensive to remove one 50 L drum of pit contents?
8. If this service were available in this area, how many drums of sludge would you have removed from your pit at Tsh 5000/drum?
9. If I came back with the service provider in one month, would you be ready to pay?
10. If no, why not?

Box 1

Huduma Ya Gulpa Ya Upakuaji Vyoo

Hii ni huduma mpya ya upakuaji vyoo ambayo tayari imeshajaribiwa Azimio. Ina sifa zifuatazo:

- Ni nafuu - mtu analipia kwa kiasi (katika pipa) cha kinyesi ambacho angetaka kiondolewe.
- Huondoa vyote maji maji na taka ngumu
- Huchukua muda mfupi tu kutoa kinyesi
- Hakuna harufu wakati wa kupakua
- Mtu hapati kuona wala kugusa kinyesi kinapokuwa kinatolewa
- Kinyesi kinapelekwa mbali baada ya kuwa kimetolewa kutoka shimoni
- Inayafikia yaliyomo shimoni bila kuvunja slabu au muundo—hii ina maana hakuna gharama za kujenga mpya au kutengeneza slabu

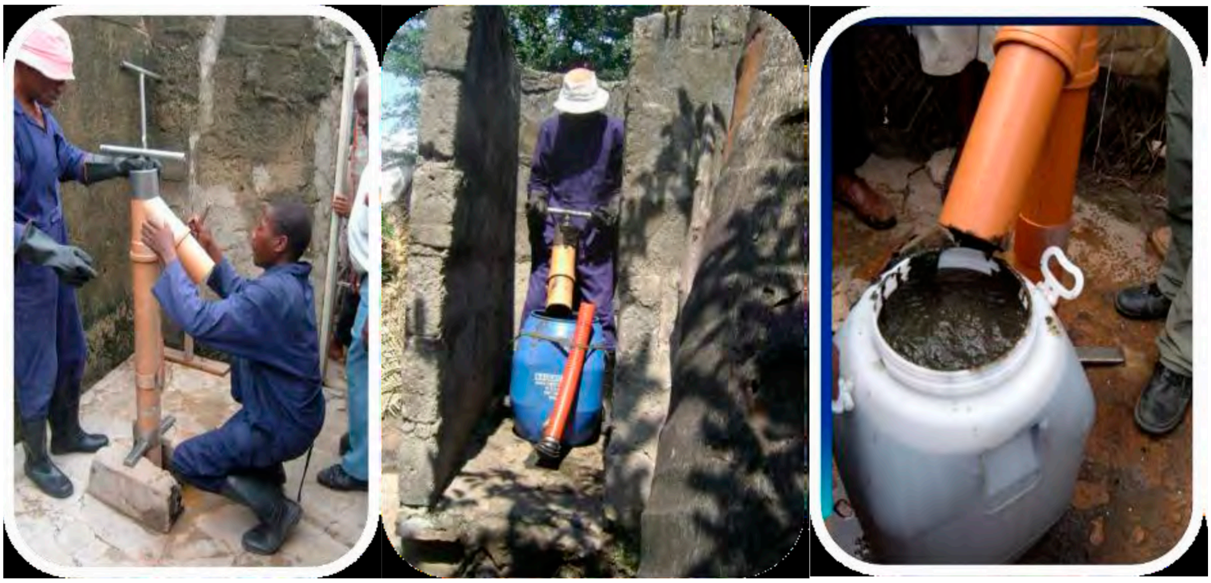

Figure S1. Gulper service operating in Azimio sub-ward in Dar Es Salaam, at the time of the survey: Pictures shown to respondents as part of the presentation.

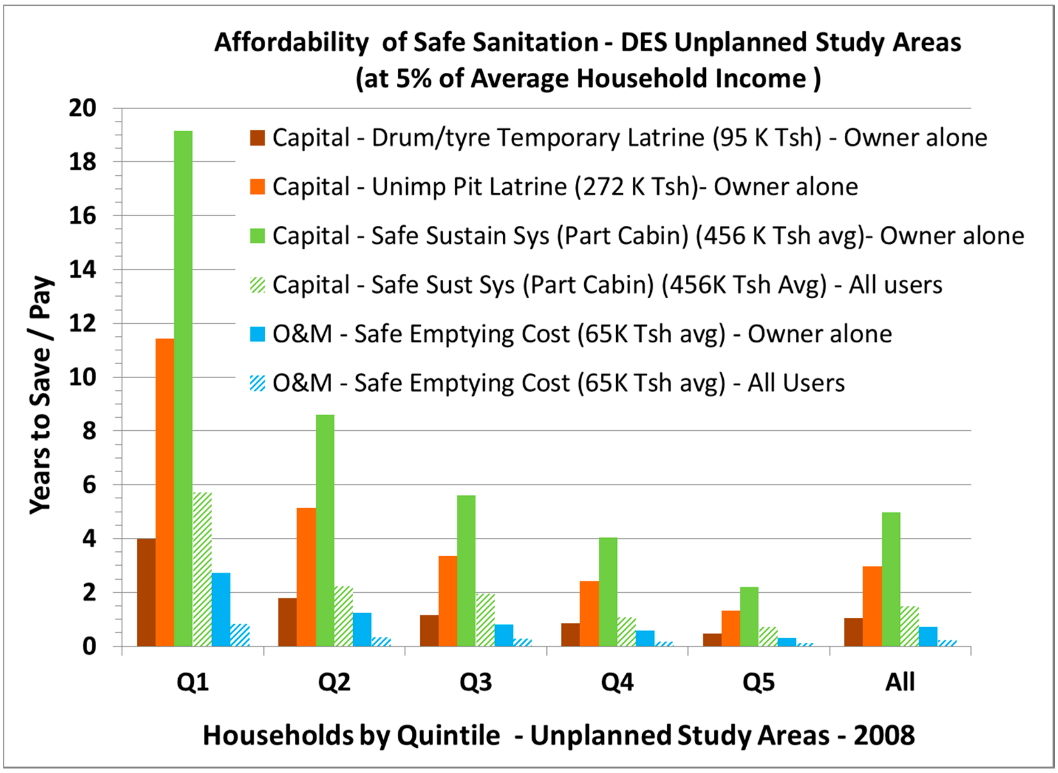

Figure S2. Time required to save for capital costs of sanitation facilities and pay for safe pit emptying service at 5% of quintile average household income in unplanned areas of Dar Es Salaam.

Table S1. Products intentionally added to the fecal sludge pit of latrines in unplanned study areas in Dar Es Salaam (2008).

**Product**	**Frequency**	**Purpose for Using the Product (Rank Order of Purpose for Product)**					
	**% (n)**	**Reduce Odors**	**Reduce Disease**	**Reduce Flies**	**Reduce Germs**	**Sink Sludge**	**Reduce Mosquitos**
Insecticide	35 (232)		√ (1)	√ (3)	√ (2)		√ (4)
Ashes	15 (97)	√ (1)	√ (3)			√ (2)	√ (4)
“Salt” **	7 (48)	√ (2)				√ (1)	√ (3)
Paraffin	6 (42)	√ (1)		√ (2)		√ (3)	√ (4)
Old batteries	6 (42)	√ (1)		√ (3)		√ (2)	
Diesel	5 (33)	√ (1)		√ (3)		√ (2)	√ (4)
Any product	53 (337)						
% of users (n)	Total of 337	47 (158)	42 (140)	29 (99)	29 (99)	17 (56)	1.4 (32)
% overall (n)	N = 660 *	24 (158)	21 (140)	15 (99)	15 (99)	8.5 (56)	5 (32)
Number of products used (per user)			Mean 1.4	S.D. 0.80	Min 1	Max 6	Median 1

* Out of 662 residences, two did not have a latrine. ** The pit additive locally referred to as “salt” is thought to be the salt-like waste by-product, calcium hydroxide ($Ca(OH)_2$), of hydrolysis of calcium carbide to produce Acetylene gas in low-tech soldering/welding operations. Use of welding “salt” waste as a pit additive to reduce pit sludge volume and control odors was reported by latrine owners in rural Benin in a 1994 study [1]. Sanitation Promotion in Developing Countries. PhD Dissertation. Civil & Environmental Engineering, UC Davis). Calcium hydroxide is also known as slaked lime and raises the pH of a saturated

solution to above 12. According to Wikipedia besides raising pH, it is used as an insecticide against crawling insects, which are killed by its touch (http://en.wikipedia.org/wiki/Calcium_hydroxide).

Table S2. Amounts paid * to empty on-site sanitation facilities in unplanned areas of Dar Es Salaam in 2008.

Row Definition	Cost of Service TSH (USD)	Cost per User Household TSH (USD)	Cost/Year: Per User ** TSH (USD)	Cost/Year: per Household TSH (USD)	Cost/Year: % Household Ann. Income
Mean overall (n = 166 to 224) (ex. sink sludge)	66,514 ($57)	6,970 ($5.93)	873 ($0.74)	3436 ($2.92)	0.3%
Median overall	40,990 ($35)	5060 ($4.31)	357 ($0.30)	1100 ($0.94)	0.08%
Min–Max	0–280,010 ($0–$238)	0–43,440 ($0–$67)	0–10,898 ($0–$9.27)	0–74,376 ($0–$63)	0%–8% ***
**By Method (mean):**					
Pit Diversion (n = 99 to 135)	71,320 ($61)	7020 ($5.97)	801 ($0.68)	3180 ($2.70)	0.24%
Vacuum Tanker (n = 30 to 40)	62,760 ($53)	7160 ($6.09)	856 ($0.73)	2650 ($2,26)	0.14 %
Flooding out (n = 19 to 26)	46,640 ($40)	4500 ($3.83)	881 ($0.75)	5080 ($4.32)	0.8%
Vacutug (n = 9 to 11)	76,930 ($65)	10,740 ($9.14)	1023 ($0.87)	1760 ($1.50)	0.11%
Manual Bucket (n = 9 to 12)	58,460 ($50)	7720 ($6.57)	1586 ($1.35)	7620 ($6.48)	0.44%
Sink sludge (n = 5)	19,500 ($17)		238 ($0.20)		

* Based on reported expenditures for the last emptying event within 10 years prior to the residential survey, adjusted to 2008 TSH values and converted to 2008 U.S. $ at TSH 1175 per U.S. $; ** Emptying cost divided by facility-specific average time to fill and current number of reported users including adults and children age 3 and over; *** 8 % is an extreme outlier.

Reference

1. Jenkins, M.W. Sanitation Promotion in Developing Countries: Why the Latrines of Benin are Few and Far Between. PhD Dissertation, Department of Civil and Environmental Engineering, University of California Davis: Davis, California, USA, 1999. Available online: https://watershed.ucdavis.edu/shed/lund/students/JenkinsDissertation.pdf (accessed on 26 February 2015).

© 2015 by the authors; licensee MDPI, Basel, Switzerland. This article is an open access article distributed under the terms and conditions of the Creative Commons Attribution license (http://creativecommons.org/licenses/by/4.0/).
